# Choosing the Right Cell Line for Acute Myeloid Leukemia (AML) Research

**DOI:** 10.3390/ijms24065377

**Published:** 2023-03-11

**Authors:** Rafał Skopek, Małgorzata Palusińska, Katarzyna Kaczor-Keller, Rafał Pingwara, Anna Papierniak-Wyglądała, Tino Schenk, Sławomir Lewicki, Artur Zelent, Łukasz Szymański

**Affiliations:** 1 Department of Molecular Biology, Institute of Genetics and Animal Biotechnology, Polish Academy of Sciences, Postępu 36A, 05-552 Magdalenka, Poland; 2 Department of Physiological Sciences, Institute of Veterinary Medicine, Warsaw University of Life Sciences-SGGW, 02-787 Warsaw, Poland; 3 Polbionica Sp. z.o.o., Aleja Prymasa Tysiąclecia 79A, 01-242 Warsaw, Poland; 4 Department of Hematology and Medical Oncology, Clinic of Internal Medicine II, Jena University Hospital, 07747 Jena, Germany; 5 Institute of Molecular Cell Biology, Center for Molecular Biomedicine Jena (CMB), Jena University Hospital, 07747 Jena, Germany; 6 Faculty of Medical Sciences and Health Sciences, Kazimierz Pulaski University of Technology and Humanities, 26-600 Radom, Poland; 7 Institute of Outcomes Research, Maria Sklodowska-Curie Medical Academy, 00-001 Warsaw, Poland

**Keywords:** AML cell lines, WHO, FAB, AML subtypes, CD markers, genetic rearrangements

## Abstract

Immortalized cell lines are widely used in vitro tools in oncology and hematology research. While these cell lines represent artificial systems and may accumulate genetic aberrations with each passage, they are still considered valuable models for pilot, preliminary, and screening studies. Despite their limitations, cell lines are cost-effective and provide repeatable and comparable results. Choosing the appropriate cell line for acute myeloid leukemia (AML) research is crucial for obtaining reliable and relevant results. Several factors should be considered when selecting a cell line for AML research, such as specific markers and genetic abnormalities associated with different subtypes of AML. It is also essential to evaluate the karyotype and mutational profile of the cell line, as these can influence the behavior and response to the treatment of the cells. In this review, we evaluate immortalized AML cell lines and discuss the issues surrounding them concerning the revised World Health Organization and the French–American–British classifications.

## 1. Introduction

Two classifications of AML subtypes have been established up till now. Historically, the first one is the French–American–British (FAB) system based on morphological and cytochemical findings [1]. According to FAB, eight types of AML can be diagnosed: AML with minimal differentiation (M0), AML without maturation (M1), AML with maturation (M2), acute promyelocytic leukemia (M3), acute myelomonocytic leukemia (M4), acute monoblastic/monocytic leukemia (M5), acute erythroid leukemia (M6), acute megakaryoblastic leukemia (M7). The FAB AML type may be distinguished based on the assessment of blood marrow and peripheral blood [2].

## 2. FAB Classification and Diagnostic Process

The FAB classification system for AML was first established in 1976 and categorized AML based on morphological and cytochemical features, such as cell morphology, immunophenotype, and clinical symptoms. The FAB classification is related to the AML diagnostic process, which begins by examining specific cytological features that allow for M3 diagnosis. If M3 is ruled out, the diagnosis proceeds to M6, characterized by at least 50% of bone marrow cells being erythroid cells and at least 30% of non-erythroid cells being blast cells. If these criteria are met, M6 is diagnosed. If they are not, the number of blasts in nucleated bone marrow cells is assessed. If at least 30% of nucleated bone marrow cells are blasts, the diagnosis of MDS is excluded, and the diagnosis proceeds to other AML types. An additional criterion for further diagnosis is the presence of at least 3% of blast cells that are positive for myeloperoxidase (MPO)/Sudan black B (SBB) staining (indicating granulocytic differentiation) or nonspecific esterase (NSE) staining (indicating monocytic differentiation). If positive blasts are present, the diagnosis is either AML M1, M2, M4, or M5. Negative results for MPO/SBB and NSE suggest either M0 or M7 or possibly acute lymphoblastic leukemia (ALL). Thus, for MPO/SBB negative cells, the diagnosis can be either M0, M7, or ALL. M0 is characterized by positive cytochemical stains in fewer than 3% of blast cells, indicating minimal evidence of myeloid differentiation. M1 is diagnosed when at least 90% of non-erythroid cells are blasts, and at least 3% of blasts are MPO/SBB or NSE positive [1,2]. M2 is characterized by more than 50% promyelocytes and myeloblasts in the bone marrow, with the total number of blasts ranging from 30% to 89% of non-erythroid cells. Additionally, for M2, the presence of less than 20% monocytic cells and more than 10% granulocytes are specific.

M4 is characterized by an increased number of immature granulocytic and monocytic cells with abnormal proportions. Myeloblasts, monoblasts, and promonocytes comprise over 20% of marrow nucleated cells. The M4eo (acute myelomonocytic leukemia with eosinophilia) is a variant of M4 with more than 5% abnormal eosinophilic cells [3,4].

**Figure 1:**
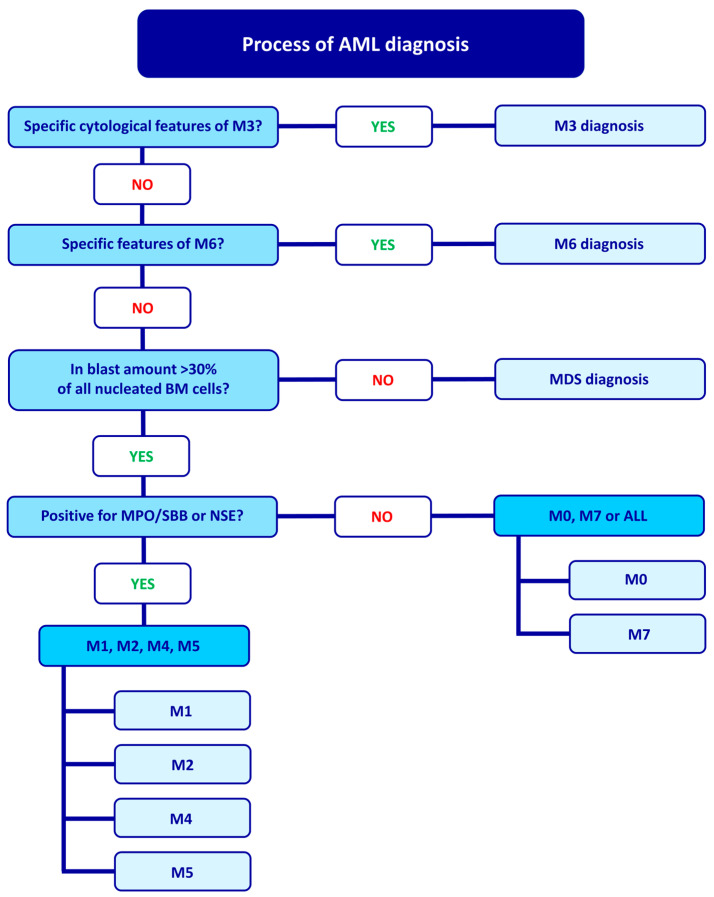
Schematic diagram representing the AML diagnosis process based on the FAB classification and the samples’ immunophenotypic, cytochemical, and morphological properties.

When monocytic cells in the bone marrow make up more than 80%, M5 is diagnosed. This subtype is further divided into M5a (acute monoblastic leukemia), in which monoblasts make up more than 80% of the leukemic cells, and M5b (acute monocytic leukemia), in which promonocytes make up more than 80% of the leukemic cells [3]. M5a is characterized by large blasts with a rounded nucleus and a very large, intensely basophilic cytoplasm. In contrast, M5b is represented by promonocytes with a rounded or kidney-shaped nucleus and a less basophilic, more granular cytoplasm with vacuoles [3]. The diagnostic procedure for AML according to the FAB classification is depicted in Figure 1.

## 3. WHO Classification

**Figure 2:**
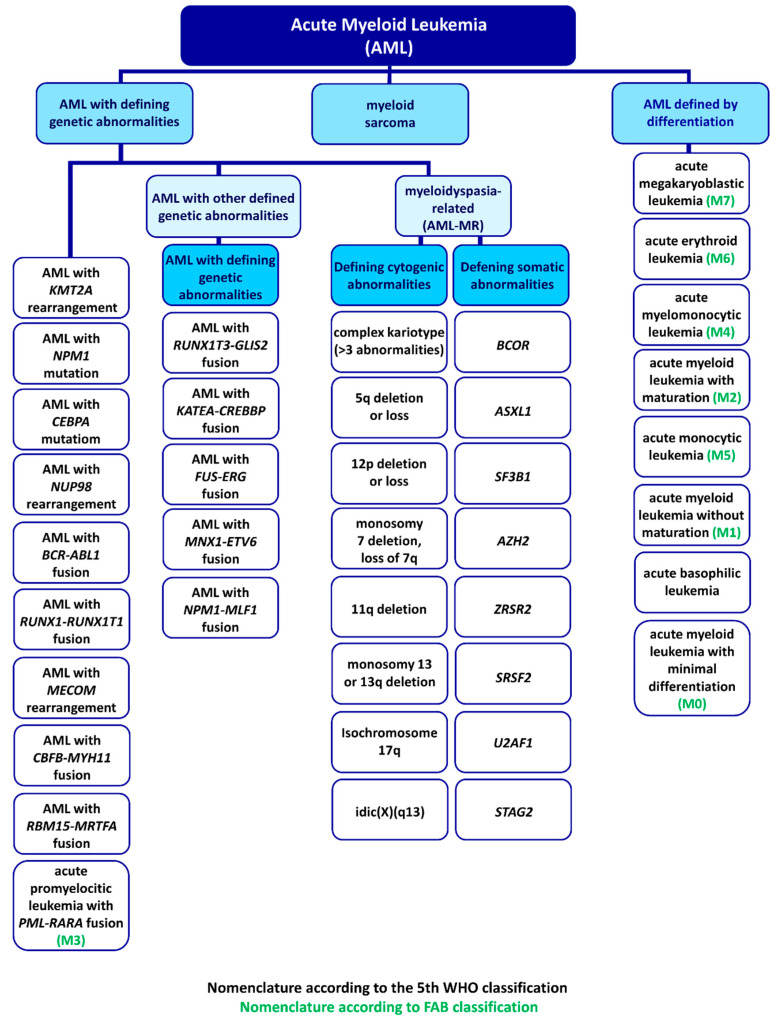
AML classification based on the fifth edition of the WHO classification [5].

The FAB classification does not take into account chromosomal and molecular features. Therefore, in 2008, the World Health Organization (WHO) introduced the fourth WHO classification system that included these additional features, a clinical history of chemotherapy and myelodysplasia-related changes, and the categories proposed by FAB. The fifth edition of the WHO classification, released in 2022, also include genetic abnormalities and divides AML into 32 subtypes based on key mutations and genetic alterations that define the clinical outcome of the disease [5]. These subtypes are depicted in Figure 2.

Before the publication of the fifth edition of the WHO classification, the FAB classification was still widely used in many countries due to a lack of funding and limited access to genetic analysis equipment [6]. However, the most recent WHO classification considers both differentiation patterns and genetic aberrations, combining the fourth WHO and FAB classifications and thus making it more accurate. In the clinical setting, the FAB classification is used first because it allows for a quick preliminary qualification of the AML type. As a result, the specific M0–M7 tags used in the FAB classification will continue to be used to describe clinical cases, even with the inclusion of the WHO classification.

## 4. AML Cell Lines and Types

Considering the complexity of AML, it is impossible to identify a single cell line to establish a universal disease model. Studies conducted in non-human animal models may not provide accurate results because of differences in AML progression across species [7]. While studies using primary samples from patients may be more similar to clinical outcomes in patients, they can be diverse due to the varied genotypes of each patient. On the other hand, studies using established cell lines are more cost-effective and reproducible but do not account for the variability of clinical features in patients. Therefore, combining studies using established cell lines with those using primary samples can provide more accurate results [7].

**Figure 3:**
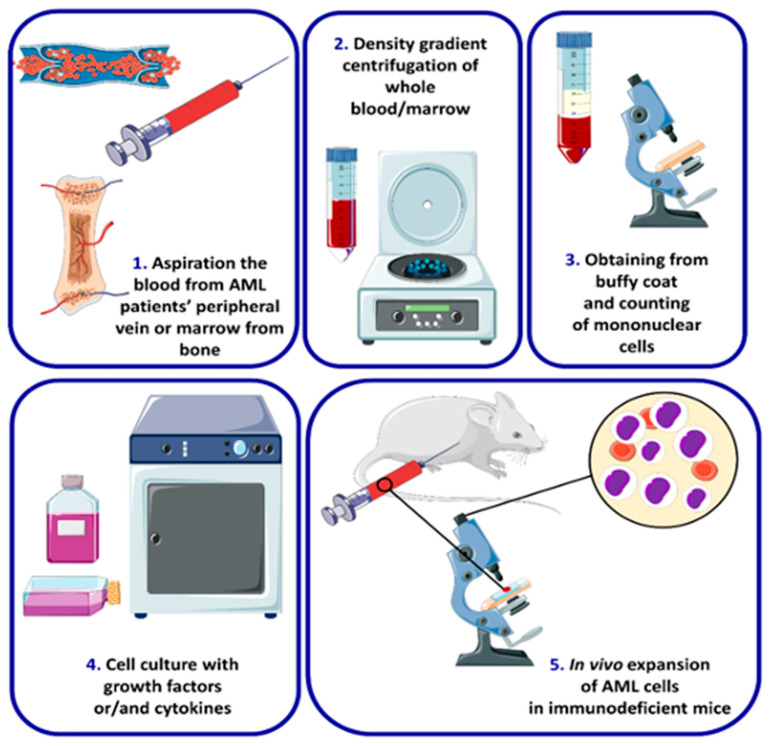
Simplified diagram of the procedure for the establishment of human leukemic cell lines. **1**. AML cell lines are obtained from human peripheral blood or bone marrow. After blood/marrow aspiration, the sample is mixed with PBS in a 1:1 or 1:2 ratio and transferred to centrifuge tubes containing gradient buffer. **2**. The obtained cell suspension is centrifugated in the density gradient buffer to obtain a buffy coat consisting of mononuclear cells. **3**. The next step is isolated cell counting and suspension of the cells in fresh medium and transferring them to the culture flask, plate, or dish. **4**. Continuous culture of the isolated cells in a selected medium supplemented with or without fetal bovine/calf serum (FBS/FCS), growth factors, and cytokines. **5**. To verify the malignant origin of these cells, they are inoculated to the immunodeficient mice. The figure was created using templates from Servier Medical Art, licensed under a Creative Commons Attribution 3.0 Unported License (http://smart.servier.com/, accessed on 27 December 2022).

Many AML cell lines with specific mutations have been established over the years. In contrast to primary samples, established AML cell lines can grow independently in tissue culture and often do not require exogenously supplied hematopoietic growth factors or other cytokines. The cell line establishment procedure is presented in Figure 3.

### 4.1. AML with Minimal Differentiation (M0)

In the M0, the leukemic cells have an agranular cytoplasm and are negative for histochemical staining with SBB and NSE but are positive for MPO in most cases. M0 can be distinguished from ALL by the presence of at least 3% of cells positive for MPO [8]. The karyotypes associated with an unfavorable prognosis in M0 include −5, del(5q), −7, del(7q), +8, and del(11q). Terminal deoxynucleotidyl transferase (TdT) activity is present in about 50% of patients with M0. Most M0 blasts are positive for at least two myeloid markers, CD13 and CD117, and may also express CD34, CD7, and HLA-DR [9,10]. To date, two cell lines derived from patients with M0, Kasumi-3, and MOLM-16 have been established.

#### 4.1.1. Kasumi-3

The Kasumi-3 cell line was derived from a 57-year-old male patient with M0. It has a few notable characteristics, including a doubling time of approximately 55–60 h in cell culture, an appearance consistent with immature lymphoblasts, and the expression of various markers, as shown in Table 1 [11]. B cell-specific, T-ALL, and NK markers were also identified in this cell line [12]. Treatment with 12-O-tetradecanoylphorbol-13-acetate (TPA) leads to monocytic maturation, but DMSO, all-trans retinoic acid (ATRA), and cytokines do not induce the expression of CD2, CD3, and CD8 [11]. Moreover, the cell line exhibits several chromosomal abnormalities such as t(3;7) (q27:q22), del(5) (q15), del(9) (q32), add(12) (p11), −8, t(2;5) (p13;q33), and add(16) q(13), +mar [11]. Since 3q27 is located near the ectopic virus integration-1 gene (*EVI1*), high expression of the *EVI1* is observed [11]. The *EVI1* is a transcriptional regulator involved in stem-like programs in hematopoietic progenitor cells and solid tumors, it is essential for self-renewal and growth, but its exact function is yet to be determined [13,14]. Elevated *EVI1* expression suggests that the Kasumi-3 may be a potential model to study the influence of BET inhibitors on the *EVI1* downregulation and its activity in AML [14].

The MECOM mutation and activation have also been observed in Kasumi-3 [12]. Therefore, Kasumi-3 not only represents the biological characteristics of M0 but also constitutes a good model for studying *EV11* and *MECOM.* The Kasumi-3 engraftment model in NSG mice was also developed. Mice were implanted with 1 × 10^6^ or 10 × 10^6^ per mouse. The amount of human CD45+ cells in peripheral blood, blood marrow, and spleen was monitored by FACS to estimate the disease progression. The Kasumi-3 cells were found in each studied variant (peripheral blood, bone marrow, spleen). Tumor burden was established sooner in higher cell concentrations. Those results show the possibility of using the Kasumi-3-NSG-mice model as an in vivo tool for evaluating therapeutics or epigenetic compounds for AML [15].

#### 4.1.2. MOLM-16

The MOLM16 cell line represents the M0 with megakaryoblastic features and was derived from the peripheral blood of a 77-year-old woman with M0 AML that had relapsed following treatment with behenoyl cytosine arabinoside, daunomycin, and 6-mercaptopurine [16]. After 13 weeks of culture with a 5637 cell line supernatant known to secrete various growth factors, including GM-CSF, IL-3, IL-6, and SCF, the MOLM16 cells began proliferating [16,17]. The MOLM16 cells exhibit a myeloid NK precursor acute leukemia phenotype of megakaryocytes (Table 1). They also express markers of the erythroid and myeloid lineage and markers of immature cells. Genetic analysis of bone marrow cells revealed the presence of chromosomal abnormalities, including add(3) (q27), add(9) (p11), −14, −18, add(19) (p13), −21, and +3mar in 16/20 and 4 [16]. The MOLM16 cells also carry unique translocations, including t(6;8) (q21;q24.3), which have been associated with poor prognosis and t(9;18) (q13;q21) whose significance is still being determined [16,18]. The MOLM16 cells express high levels of PIM and receptor tyrosine kinase gene (FLT3), making them (and the MV4-11 cell line) potentially useful for studying the effects of inhibitory compounds on PIM/FLT3 signaling [19]. In addition, the MOLM16 cells contain the JAK2 V617F mutation in the JH2 pseudokinase domain of the JAK2 gene, frequently observed in MDS and myeloproliferative disorders (MPD) and has been linked to leukemogenesis [20]. This suggests that the MOLM16 cells and other AML cell lines such as HEL, MB-02, MUTZ-8, SET-2, and UKE-1 may be useful for creating a panel of cell lines to study MDS and MPD in the context of JAK2 V617F. The MOLM16 cells also show the deletion of the *PMS2* and *RSPH10B2* genes [21].

### 4.2. AML without Maturation (M1)

M1 is a subtype of AML that occurs in approximately 10–15% of all cases of AML. It is less common in children [3]. This subtype of AML is characterized by various chromosomal abnormalities, including t(9;12), t(11;19), t(14;17), der(12), t(12;17), and der(16) t(16;20) [3]. In M1 AML, the myeloid cells in the bone marrow are typically medium-sized and have a high nucleus-to-cytoplasm ratio. The nuclei are round and have immature, diffuse chromatin with one or more distinct nucleoli. The blasts in this subtype express certain myeloid antigens, including MPO, CD13, CD33, CD117, and CD34low [4]. There have been reports of a mutual exchange between the *D12S158* gene at 12p13.3 and the *MYH11* gene at 16p13 in M1.

#### 4.2.1. CTS

The CTS cell line was derived from the peripheral blood of a 13-year-old girl with M1 acute myeloid leukemia in relapse. The cells are derived from an immature hematopoietic cell with multilineage capabilities [22]. The cells have a round or ovoid shape and large nuclei with few nucleoli and basophilic cytoplasm. The CTS cells have a 72-h doubling time in medium supplemented with 10% FCS. The cells contain DNA rearrangements in the immunoglobulin heavy and light κ chain genes and deletions in the T-cell receptor δ1 gene. The CTS cells have a diploid karyotype with a t(6;11) (q27;q23) chromosomal translocation. In addition, the CTS cells have a *KMT2A* (previously known as *MLL*) rearrangement, resulting in the expression of a KMT2A/AF6 fusion transcript. This alteration makes the CTS cells useful for studying *KMT2A* interactions. In terms of proliferation, the CTS cells are unresponsive to IL-2, IL-3, IL-6, IL-11, GM-CSF, G-CSF, erythropoietin (EPO), and SCF but can be induced into T-cell, B-cell, erythroid, or megakaryocytic lineages with GM-CSF and G-CSF, making them useful for differentiation studies. Finally, the CTS cell line may be useful for studying oncogenesis associated with *KMT2A* and 11q23 abnormalities and for studying pluripotent stem cells [22].

#### 4.2.2. UoC-M1

The UoC-M1 is a hypodiploid cell line with *KMT2A* rearrangement and megakaryoblastic features derived from a 68-year-old patient with AML [23]. The UoC-M1 is similar to the ELF-153 cell line. Both lines are growth factor-independent, have monosomy 7, a loss of 5q, and a lack of erythroid markers. However, the ELF-153 cell line is CD38 negative and carries a t(12;14) (p11.1;q11.1) chromosomal translocation, and some cells are polyploid up to 32 N. In contrast, UoC-M1 has three segments of chromosome 9 interspersed with three segments of chromosome 11, resulting in *KMT2A* duplication. In addition, UoC-M1 has four copies of *KMT2A*, leading to higher levels of KMT2A transcript than other cell lines.

#### 4.2.3. KG-1

The KG-1 is a cell line established from a 59-year-old man with erythroleukemia that progressed to acute myelogenous leukemia. The cells are mainly at the myeloblast or promyelocyte stage of maturation. In the presence of DMSO, the cells can differentiate into granulocytes, but they are unresponsive to ATRA [24]. KG-1 cells express wild-type DNA methyltransferase 3 alpha (DNMT3A) and KMT2A and are often used as a control line to study the role of DNMT3A in leukemia development [25,26]. KG-1 cells are frequently used in toxicology and cancer immunology research. KG-1 myeloblasts can differentiate into macrophages in the presence of phorbol esters and respond to CSF. The fact that the KG-1 was initially isolated from a patient diagnosed with erythroleukemia contributes to the misclassification of this cell line. Indeed, initially, many publications classified the KG-1 as M6, but recent publications suggest otherwise. Interestingly, the KG-1 was also suggested to be M0 [27] or M2 [28]. Considering current data, the KG-1 should be classified as myeloblasts with minimal maturation and no ability to differentiate—M1 [29,30].

The KG-1a is a less differentiated cell line isolated after 35 passages from KG-1 [31]. The KG-1a cells are morphologically, cytochemically, and functionally less mature than KG-1 cells and are mainly promyeloblasts. Compared to the parental line, KG-1a cells are unresponsive to CSF and do not express HLA, contributing to increased resistance to differentiation-inducing drugs [31]. As a chemotherapy-resistant cell line, KG-1a is used for cytotoxicity studies of new AML therapies. For example, the effectiveness of arsenic trioxide (ATO) in M3 treatment has been demonstrated using KG-1a cells. ATO has a cytotoxic effect on KG-1a, reducing cell proliferation and inducing apoptosis by altering the expression of genes related to these processes. In addition, ATO significantly reduces the expression of MYC, PCNA, and MCM7 at the gene and protein levels in KG-1a, suggesting its potential use in studying these genes [32]. The KG-1 and KG-1a have an identical karyotype of 47, X.Y.Y, 7q−, 8p+, i2p+, −20, +MAR-i, +MAR-2 [31]. The KG-1a is nowadays classified as M1 [33] or even M0 [28,30,34]. Yet the affiliation of KG-1 and KG-1a should be explicitly determined.

KG-1 and KG-1a are challenging to differentiate in vitro, making them valuable for studying potential drugs that promote differentiation, such as differentiating CD34+ progenitor cells into dendritic-like cells [35,36].

#### 4.2.4. K-562

The K-562 cell line is a well-known cell line that has been widely used in research for many years. It was initially obtained from the bone marrow of a 53-year-old patient with chronic myeloid leukemia (CML). The K-562 was originally classified as the M6, relying on its highly undifferentiated state and surface membrane properties; however, the newest reports show that K-562 is, in fact, characteristic for the M1 [37,38]. The K-562 cells are aneuploid and have the Philadelphia (Ph1) chromosome and a long acrocentric marker. The Ph1 chromosome is characterized by the deletion of the long arm of chromosome 22 (del(22) (q12)). In contrast, the long acrocentric marker results from an unbalanced reciprocal translocation between chromosome 17 and the long arm of chromosome 15 [39,40]. The K-562 cells also have the *BCR-ABL1* fusion gene, resulting from a balanced translocation between chromosomes 9 and 22 [41]. Mutations of *BRCA1*, *ASXL1*, *MLH1*, *BIRC6*, *AKT3*, *TERT*, and *FANCC* are also present in this cell line, which suggests the potential application of K-562 in studying those genes [42]. In addition, the K-562 cells are deficient in the p53 protein, which plays a crucial role in cell cycle arrest and survival. The inhibition of Aurora kinase leads to apoptosis in K-562 cells by regulating the expression of pro-apoptotic proteins such as Bax and p53 and decreasing the level of BCL-2 [40,43]. The stress response in K-562 cells has also been studied in relation to Sirtuin-1, which regulates cellular stress, metabolism, and autophagy-associated deacetylase activity [44].

The K-562 cells are a pluripotent and highly undifferentiated cell line, which makes them a valuable model for studying the expression of erythroblastoid properties and the synthesis of hemoglobin during erythroid differentiation. They have also been used in in vitro natural killer assays because they can induce the production of fetal or embryonic hemoglobin in the presence of hemin T [45]. When treated with different substances, the K-562 cells can differentiate into erythrocytic, granulocytic, monocytic, or megakaryocytic lineages. For example, imatinib and cyclosporine A can cause erythrocytic differentiation, while TPA can induce megakaryocytic maturation [46]. In addition, phorbol esters can stimulate the differentiation of K-562 cells along the megakaryocytic lineage and can also substitute for monocyte-accessory cell function [47,48].

### 4.3. AML with Maturation (M2)

The AML with maturation accounts for approximately 25% of adult AML cases. One of the most common chromosomal translocations seen in the M2 subtype AML is t(8;21) (q22;q22), which involves rearrangements of the *RUNX1* (previously described as *AML1* or *CBFA2*) [49] and *RUNX1T1* (previously described as *ETO*, *AML1T1*, *CBFA2T1*) [49] genes and can lead to the differentiation of leukemic cells into mature neutrophils and eosinophils, and in some cases, myeloblastomas [50]. The *CEBPA* gene is often mutated in AML with maturation, occurring in up to 20% of patients [51], and this mutation promotes cell differentiation and apoptosis [52]. Another mutation commonly found in the M2 is t(6;9) (p23;q34), associated with poor prognosis and distinct clinical and morphologic features. This translocation results in the formation of the *DEK-NUP214* fusion gene, which encodes a messenger RNA involved in leukemogenesis [53] and is associated with a high prevalence (70%) of *FLT3*- internal tandem duplications (ITD) mutation. This prevalence is higher than in other AML types. The *FLT3* gene plays a role in hematopoietic cell proliferation and differentiation. Patients with the *FLT3*-ITD mutation often have higher white blood cell counts and bone marrow blasts but lower complete remission rates. This mutation is considered one of the most lethal in AML [53,54,55].

#### 4.3.1. Kasumi-1

The Kasumi-1 is a widely studied cell line derived from a 7-year-old boy with acute myeloid leukemia (AML) and the t(8;21) chromosomal translocation, which is associated with core binding factor leukemia (CBFL). It carries the *RUNX1-RUNX1T1* fusion gene [12] and a ligand-independent c-kit mutation (Asn822Lys) [56]. The Kasumi-1 also has several other genetic abnormalities, including trisomy of chromosome 10, *TP53* mutation, monosomy of chromosome 13, and the retinoblastoma gene (*Rb-1*) located on 13q14 [11]. This cell line expresses myeloperoxidase and can differentiate into neutrophils and macrophages after treatment with IL-5 or TPA, respectively. It also expresses CD34, suggesting it originated from an early myeloid stem cell. The Kasumi-1 forms spontaneous tumors in culture, with 10–20% of cells adhering to the culture flask and forming aggregates [11]. Tumor formation ability shows the similarity of Kasumi-1 to the clinical outcome of AML with t(8;21).

The Kasumi-1 has several characteristics that make it a model for studying AML. It has a good response to chemotherapy, with a high remission rate and prolonged survival, and it reflects the M2 subtype seen in younger patients. It also exhibits solid tumor formation with myeloblastoma features and displays abnormal leukemic cell maturation, low neutrophil alkaline phosphatase activity, and Auer rods in blasts and mature granulocytes. It can differentiate into granulocytic and macrophage lineages and may help study the molecular, morphologic, and immunophenotypic features of AML with t(8;21). Additionally, the Kasumi-1 is helpful for studying the role of the *RUNX1-RUNX1T1* fusion oncogene in myeloid differentiation [11,56].

#### 4.3.2. Kasumi-6

The Kasumi-6 is a cell line derived from the blast cells of the bone marrow of an elderly patient who experienced a relapse of M2 [57]. Like the Kasumi-1, it has a trisomy of chromosome 10 and a TP53 mutation. However, it has unique characteristics, including FLT3-ITD and CEBPA mutations [12,57]. Kasumi-6 is resistant to several agents that can induce differentiation, including DMSO, ATRA, 1,25D3, G-CSF, and GM-CSF, but not TPA. The loss of CEBPA expression contributes to this resistance to differentiation agents. CEBPA loss makes Kasumi-6 a suitable model for studying differentiation. There is interest in using epigenetically active agents to promote differentiation in this cell line by overcoming the dominant-negative effect of the mutant CEBPA [57].

#### 4.3.3. SKNO-1

The SKNO-1 is a cell line derived from the bone marrow of a 22-year-old male with M2 acute myeloid leukemia and the t(8;21) (q22;q22) chromosomal translocation. The patient’s leukemia was characterized by the presence of the *RUNX1*/*RUNX1T1* fusion gene and became resistant to chemotherapy after acquiring monosomy of chromosome 17 [37,58]. SKNO-1 is cultured as a GM-CSF-dependent cell line and carries the same t(8;21) and monosomy 17 as the cells of origin [58]. The *RUNX1* gene on chromosome 21 is rearranged in this cell line, leading to the expression of the *RUNX1*-*RUNX1T1* fusion transcript. The SKNO-1 also has *p53* gene overexpression and mutation, suggesting it may be used for studying the molecular mechanisms of leukemogenesis in AML with t(8;21). It has also been used to study the effects of a co-repressor complex containing histone deacetylase (HDAC) inhibitors [59].

#### 4.3.4. HL-60

The HL-60 cell line was initially derived from the peripheral blood of a 36-year-old patient diagnosed with M3 acute myeloid leukemia [60]. However, karyotyping later revealed the absence of the t(15;17) translocation and deletion of the p53 gene, leading to the classification of the HL-60 as M2 [60,61,62]. The HL-60 was initially cultured in a medium obtained from human embryonic lung cells and achieved independent growth during subsequent passages. These cells are known to have a doubling time of 36–72 h, although this may vary depending on the source [60,63]. The HL-60 cells can form colonies in semisolid and produce chloromas (subcutaneous myeloid tumors) in nude mice [60]. They stain positive for myeloperoxidase and SBB and negative for alkaline phosphatase [63]. The predominant cell type in fresh and cultured cells was a neutrophilic promyelocyte with nuclear and cytoplasmic asynchrony. They are also known to spontaneously differentiate beyond the promyelocyte stage into myeloblasts, myelocytes, and granulocytes. They can be induced to differentiate into granulocytes or macrophages through butyric acid, triethylene glycol, N_1_N-dimethylformamide, N_1_N_1_-dimethylacetamide, and 1.3% DMSO stimulation [45,60]. Differentiation into macrophages is induced by phorbol esters and results in the production of adhesive molecules, long pseudopodia development, NADase, NSE production, and Fc receptors expression [45,64]. High differentiation capability suggests using the HL-60 in the studies of myeloid cell differentiation into the granulocytic and mononuclear lineage.

Some difficulties have been reported in differentiating the HL-60 cells with ATRA or DMSO, although this is still an area of ongoing research [65]. Our experience shows that HL-60 needs to be cultured in low concentration, subcultured (with the replacement of culture media) every third day to a concentration 1 × 10^5^ per milliliter to exhibit differentiation potential. Moreover, the differentiation ability of the HL-60 appears around the second week of culture (unpublished data). The HL-60 cells are aneuploid with characteristic deletions of chromosomes 5, 8, and X, as well as the addition of a marker resembling a D-group acrocentric and a submetacentric marker [63]. They also exhibit phagocytic activity, chemotactic response, the production of superoxide, and the reduction of the NBT dye [45,60]. The TdT was also not detected in this cell line [45]. It was shown that in the DMSO-induced HL-60, the lactoferrin gene, although present, is not transcribed [66]. In the undifferentiated HL-60, ectopic expression of transferrin and insulin receptors is present and contributes to growth-in-suspension ability [60].

These characteristics make the HL-60 cells useful in studying myeloid differentiation, the influence of chemotherapeutics, and the effects of plant secondary metabolites on anti-proliferation, pro-apoptosis, cell cycle arrest, and differentiation [61]. Additionally, the miRNA expression profile of the HL-60 cells undergoing ATRA-induced granulocytic differentiation has been studied using bioinformatic tools and in vitro experiments, offering the opportunity to examine the oncogenic and tumor-suppressive functions of various miRNAs in the context of leukemogenesis [67].

#### 4.3.5. PLB-985

The PLB-985 is a cell line derived from the peripheral blood of a patient with refractory myeloblastic leukemia. It is characterized by its karyotype, which carries no recognizable translocations and no specific chromosome markers and is a diploid cell line enriched in the myeloblast population. The PLB-985 cells can mature into granulocytic, monocyte, or macrophage cells in the presence of inducing agents such as phorbol esters [68]. They can also undergo granulocytic maturation in the presence of DMSO, ATRA, or dibutyryl cyclic adenosine. Although there have been reports of contamination with HL-60, PLB-985 is generally considered a subclone of HL-60 with distinct properties, such as differences in gene expression and the number of neutrophil surface markers, including differences in IL-8 and cytokine receptor CXCR1 [69,70]. The PLB-985 is commonly used as a model for neutrophil differentiation, expressing CD11 and surface antigens specific for neutrophils, including the formyl peptide receptor 1 (FPR1). It is a unique model that may be useful in the study of cellular and molecular mechanisms involved in the proliferation, maturation, and differentiation of leukemic cells towards the granulocyte/monocyte/macrophage lineage and is particularly well suited for the study of the maturation of immature cells at an earlier stage than the HL-60 [65,68].

### 4.4. Acute Promyelocytic Leukemia (M3)

Acute promyelocytic leukemia (M3) accounts for 5–10% of all AML cases and is associated with a recurrent, balanced translocation between chromosomes 15 and 17 t(15;17) (q22;q12) [71,72]. Translocation of *RARA* on chromosome 15 and *PML* on chromosome 17 leads to the generation of fusion oncogene *PML::RARA.* The protein expressed from thereof is an abnormal version of RAR, which after recruitment of HDAC, leads to chromatin condensation, cell cycle arrest, cell differentiation block, and the promotion of proliferation [73,74]. Moreover, abnormal *PML-RARA* binds HDAC with a higher affinity than wild-type *RARA*, thus promoting leukemogenesis [75]. *PML-RARA* or other *RARA* hybrids are characteristic of all M3 cell lines.

Until now, many cell lines have been reported to be derived from patients with M3, yet it is essential to emphasize that the FAB classification is not based on genetic abnormalities but on cytological analysis and clinical signs of the patient. For example, the HL-60 lacks t(15;17), while the NB-4 does possess this mutation [41]. Thus, the HL-60, classified initially as M3, turns out to be a different AML type (M2) [41].

#### 4.4.1. NB4

The NB4 is an M3 cell line derived from a patient with a second ATRA-resistant relapse and is considered the only genuine M3 cell line [41]. The NB4 contains two t(15;17) chromosomes, which seem unique compared to other cell lines used in research because it carries other cytogenetic abnormalities besides the t(15;17). The NB4 was established on a cytokine-producing monolayer but grew later in a cytokine-depraved medium. The NB4 cell line has high differentiation capacity and can be differentiated into monocytes or granulocytes (mainly into neutrophils). All-trans retinoic acid (ATRA) induced terminal neutrophil maturation of the NB4. On the other hand, TPA or DMSO induces maturation into monocytes and granulocytes, respectively [76].

A few ATRA-resistant M3 cell lines have been established from NB4. The two-step continuous selective pressure of all-trans-retinoic acid generated the NB4-RAr (also named NB4-R1, NB4-LR1) cell line [77]. The NB4.306 cell line was generated by low-dose irradiation treatment (300 cGy) followed by selective ATRA pressure. The NB4.007/6 cell line, on the other hand, was established from the NB4.306 with selective pressure of ATRA and without previous radiation treatment [78]. The lack of radiation treatment resulted in obtaining the cell line morphologically similar to parent the NB4 and growing similarly to the NB4.306 at 10^−6^ M ATRA. It is suggested that compared to the wild-type NB4, the NB4.306 and the NB4.007/6 are 300 and 70 times less sensitive to ATRA [78]. Comparing both ATRA-resistant and ATRA-prone cell lines provide an excellent model for studying the induction mechanism of M3 cell maturation and ATRA resistance mechanisms and may be used as a tool for new therapeutic drug screening [77,79].

#### 4.4.2. PL-21

The PL-21 is a cell line derived from the peripheral blood of a 28-year-old female diagnosed with mediastinal granulocytic sarcoma terminating in M3 [41]. Interestingly, the t(15;17) was not identified in the PL-21 [41,79]. The PL-21 is considered to have features associated with monocytic cells [41]. The PL-21 also harbors *KRAS* and wild-type *p53* (mutation in *p53* was not detected) [80,81]. Sixty-nine AML-derived cell lines were screened for the presence of the *FLT3* mutation. Only four, including the PL-21, showed ITD of *FLT3*. Moreover, the PL-21 displayed a mutated and wild-type version of the gene [20]. This suggests that the PL-21 has a potential application as a model for studying *p53*, *FLT3*, and *KRAS* in leukemia.

#### 4.4.3. UF-1

The UF-1 is derived from the peripheral blood of a 30-year-old woman diagnosed with M3. Unlike most AML cell lines, no growth factors or feeder cell layers were used to establish this cell line. The patient relapsed, and ATRA was administered after the relapse, with no differentiation effect, resulting in clinical resistance to ATRA [82]. Other identified abnormalities are 46XX, add(ll(q44), add(6) (q12), add(7) (q36), t(15;17), (q21;q21). UF-1 shows the normal sequence in the *RARA* gene, while, e.g., HL-60 has a point mutation in codon 411 (C to T substitution) [82]. The UF-1 resistance to ATRA-induced maturation resulted from a mutation in the PML-RARA fusion gene. This mutation alters the amino acid crucial for retinoic acid binding, thereby decreasing the ligand-binding activity of the PML/RARa protein. This genetic alteration is suggested to be responsible for clinically acquired resistance to ATRA in M3 patients [83]. Thus, the UF-1 is suggested to be the first established M3 cell line with documented spontaneous ATRA-resistance [82]. This cell line may be used as a model for research concerning the block of cell differentiation and mechanisms of retinoic acid resistance. Another use may be the screening of therapeutic drugs to overcome retinoic acid resistance. Nonetheless, UF-1 has not been mentioned in many publications since 2004 [82,84,85,86,87,88,89,90].

#### 4.4.4. HT93

Similar to the UF-1, the HT93 did not require a feeder layer for the establishment and started growing in cell culture without hematopoietic growth factors (Sun et al., 2004). The HT93 was derived from a patient who relapsed twice but had not been administered ATRA in contrast to the NB4 and the UF-1. This cell line can differentiate into eosinophils or neutrophils in response to ATRA and cytokines. It also carries t(15;17) and t(1;12), leading to *PML-RARA* and *ETV6-ABL2* (previously described as *ETV6-ARG*) fusion genes. The HT93 has the *Arg611Trp* mutation in *PML-RARA*, decreasing ligand-dependent transcriptional activity and retaining a dominant-negative effect on normal RARα [83]. The presence of the *ETV6-ABL2* fusion gene contributes to autonomous cell growth and compensates for the lack of hematopoietic growth factors [91].

Moreover, the HT93 has a wild-type *TP53*. In comparison, the most popular M3 cell line, the NB4, has a *TP53* mutation. That creates an opportunity to study mutated and wild-type *TP53* in NB4 and HT93. HT93 cells should be a useful model in analyzing the effects of hematopoietic cytokines on the proliferation, differentiation, and maturation of hematopoietic progenitors [92].

#### 4.4.5. AP-1060

The AP-1060 cell line was established from a multiple-relapse patient clinically resistant to both ATRA and ATO [46]. This cell line is characteristic for t(15;17) and unique t(3;14)**.** The AP-1060 contains a *PML-RARa*Pro900Leu mutation at the same site as ATRA-resistant NB4 cell lines but accompanied by leucine instead of serine (*PML-RARa*Pro900Ser) [93]. This mutation seems to provide the AP-1060 with higher ATRA resistance than wild-type NB4 cells but is still lower than the NB4-ATRA resistant cell lines. The AP-1060’s partial resistance to ATO is suggested to be connected with obtaining a cell line from the specimen’s bone marrow after disease relapse. Yet, the reason for the resistance to ATO remains uncertain [46]. The growth and survival of the AP-1060 is cytokine-dependent, which is unique for M3 cell lines. The establishment of this cell line was composed of two stages: first, the generation of G-CSF-dependent cell strain (AP-1060S) and second, its mutagenesis with ethylnitrosourea resulting in the generation of the immortalized cell line with broadened responsiveness to hematopoietic growth factors—the AP-1060 [46]. The AP-1060 may be valuable for AML progression research focused on the resistance of M3 to ATRA and ATO. The AP-1060 line seems unique in contrast to other M3 cell lines. The mutated *PML-RARa* involved in ATRA resistance may be useful in investigating ATRA-targeted gene regulation leading to terminal differentiation. Moreover, those cells possess a more mature phenotype than the NB4 cells, providing a research model for later phases of neutrophil maturation in M3 [46]. The AP-1060 is also believed to be the first cell model possessing *ETV6-NTRK3* (EN) gene fusion found in M0, M2, highlighting its use not only in leukemias but also in solid tumors [94].

### 4.5. Acute Myelomonocytic Leukemia (M4)

Acute myelomonocytic leukemia (M4) comprises approximately 15–20% of all cases of AML [4]. Additionally, in this type of AML, the prevalence of ten-eleven translocation 2 (*TET2)* is up to 50% in M4 cases, which significantly differs from MDS and other AML types characterized by the presence of 10% to 20% *TET2* mutation. The inactivation of mutations throughout *TET2* leads to increased HSC self-renewal, altered myeloid differentiation, and increased hyper-methylated epigenetic pattern [95]. The frequency of RAS mutations is also higher in M4 and M5 than in other AML types [96]. This suggests choosing one of the M4 cell lines to study *TET2* and *RAS*.

#### 4.5.1. OCI-AML2 and OCI-AML3

The OCI-AML2 line was derived from the peripheral blood of a 65-year-old man with AML. Cells express *DNMT3A* with the *R635W* mutation [97]. DNMT3A catalyzes the DNA methylation process de novo. Somatic mutations in the *DNMT3A* occur in approximately 20% of patients with AML.

The OCI-AML3 was established from the peripheral blood of a 57-year-old man with M4. The line contains the most common and well-characterized type of *DNMT3A* mutation, the *R882* dominant and negative mutation causing reduced methyltransferase activity. In addition, the OCI-AML3 harbors the nucleophosmin (*NPM1*) gene mutation type A. The *NPM1* mutations are the most common genetic alteration in adult AML cases, accounting for about 30% of the total cases, contributing to the use of this cell line [98].

The OCI-AML2 and the OCI-AML3 are the only known human cell lines with the *DNMT3A* mutation. Therefore, they are mainly used as a model to understand the role of *DNMT3A* in leukemogenesis and to correlate drug response with *DNMT3A* mutation status in different genetic backgrounds of AML [97]. Both lines are used as a metabolic model in AML. They were used, among others, to determine the effect of ascorbate and buformin on the metabolic activity of AML cells. Studies confirmed the clinical assessment of the efficacy of the combination of these compounds in treating refractory cases of AML [99]. Bioenergetic analysis on OCI-AML2 and OCI-AML3 lines showed that *AMPK*/*PERK* activation by GSK621 induces mitochondrial apoptosis in leukemic cells [100]. The OCI-AML2 and the OCI-AML3 are used both in in vitro studies and as leukemia xenograft models to test the effectiveness of new cancer therapies.

It is essential to mention that AML cell lines named OU-AML1–8 were developed [101]. However, research in 2003 showed all eight derived lines were contaminated with the OCI-AML2 [69]. Thus, considering research results performed on these cell lines, caution is advised.

#### 4.5.2. MUTZ-11

The MUTZ-11 line was derived from a 60-year-old woman diagnosed with AML (after 2 years since MDS diagnosis). The MUTZ-11 is constitutively cytokine-dependent, with proliferation requiring cytokines provided externally [102]. The MUTZ-11 expresses only the mutated allele FLT3 ITD and KMT2A partial tandem duplication (PTD) [49,102]. *FLT3* is necessary for early hematopoiesis, and *KMT2A* is often involved in acute leukemia development. Both mutations of this gene occur in 20% and 5% of AML cases, respectively. Therefore, the MUTZ-11 can be used to study the role of *FLT3* in leukemia pathogenesis. This line was also used as a positive control in a study on the role of *FLT3* in pancreatic cancer, suggesting use as a control also in solid tumor research [103]. Research using the MUTZ-11 showed the sensitivity of cell lines with the *KMT2A* PTD mutation to inhibition of DOT1-like histone lysine methyltransferase (DOT1L), the only known histone 3 lysine 79 methyltransferase (H3K79), which is essential for the survival of and proliferation of *KMT2A* mutated cells [104,105].

#### 4.5.3. MUTZ-8

The MUTZ-8 was established from a 63-year-old woman with AML (25 years since MDS diagnosis) [106,107]. The line carries a unique karyotype, the deletion of the 5q31 AML/MDS region caused by the t(5;11) non-reciprocal translocation [106]. The MUTZ-8, like the MUTZ11, are cytokine-dependent. The cytokine response profiles showed that, among others, G-CSF-, GM-CSF-, IL-3-, M-CSF-, and SCF stimulated cell growth, whereas TGF-β1, TNF-α, and TNF-β induced cell growth inhibition [106]. Screening on 79 cell lines showed that the MUTZ-8 is one of five cell lines with the JAK2 V617F mutation. It is a common somatic mutation in exon 14 in MPD [108]. The MUTZ-8 was used as a model to confirm that the suppressor of cytokine signaling 2 (SOCS2) gene is the antagonist not only of wild-type JAK2 but also of JAK2 V617F [109].

#### 4.5.4. MUTZ-3

The MUTZ-3 was established from the peripheral blood of a 29-year-old man with AML [110]. Similar to the MUTZ-8 and the MUTZ-11, the MUTZ-3 are constitutively dependent on cytokines. Upon stimulation with GM-CSF and IL-4, the MUTZ-3 reduces CD14 levels and exhibits features of CD34-derived precursors of dendritic cells, which play a fundamental role in stimulating lymphocytes as antigen-presenting cells (APC). The MUTZ-3 dendritic cells exhibit a full range of functional antigen processing and presentation pathways [109,111]. In addition, they are a ready and standardized source of allogeneic dendritic cells to generate cytotoxic T cells for therapeutic adoptive transfer strategies [112]. Therefore, the MUTZ-3 is an adequate model for dendritic cells myeloid differentiation, showing all cell maturation transitions. Moreover, the described cell line can provide dendritic cells with phenotypic and functional properties necessary for the in vivo induction of cytotoxic T cell-mediated immunity and, therefore, a suitable candidate for dendritic cell differentiation and clinical cancer vaccine studies [113]. The MUTZ-3 cell line was also selected as a research line for tests with the human cytokine-thymic stromal lymphopoietin (TSLP). TSLP induces the growth and prevents the apoptosis of these cells, which is neutralized by adding the antibody to IL-7, indicating that TSLP binds at least part of the IL-7 receptor. Interestingly, the MUTZ-3 was the only one of the 10 cell lines tested that was TSLP and IL-7 responsive [114]. Overall, MUTZ-3 is a valuable model for studying dendritic cell function and biochemistry [115].

#### 4.5.5. ME-1

The ME-1 was established from the peripheral blood of a 40-year-old man with M4 with eosinophilia at the second relapse [37]. The ME-1 carries in(16) (p13q22) resulting in CBFB-MYH11 fusion [37]. This cell line is useful in studying inv(16). It is difficult to use the majority of commercially available cell lines because of the lack of this specific alteration in the majority of them. ME-1 cell line is a unique exception [116].

The ME-1 (with Kasumi-1 and MOLM13) has been shown to require *RUNX1* expression for proper growth and survival [117,118,119], thus suggesting the possible use of this cell line to study RUNX1 expression. This cell line was also used to study tribbles proteins: trb-1 and trb-2. The study showed that the trb-1 and the trb-2 can inhibit JNK activation associated with the Bcl-2 Ser70 phosphorylation, thus maintaining the anti-apoptotic activity of Bcl-2 [120]. In AML, MAPK-JNK signaling and the repression of trb-1 or trb-2 lead to increased cell survival and resistance to therapeutics [120,121]. This suggests a potential use of ME-1 in studies regarding JNK, JAK, Bcl-2, and tribble proteins research.

### 4.6. Acute Monoblastic and Monocytic Leukemia (M5)

Acute monoblastic and monocytic leukemia (M5) accounts for approximately 15% of all AML cases and is most common in younger individuals. In the M5 chromosomal translocation involving *KMT2A* at 11q23, including t(8;16) (p11;p13), t(9;11) (p22;q23), t(10;11) (p13;q23), t(11;19) (q23;p13) and others are frequent [122]. Similar to M4, in M5, *KMT2A* translocation is found on a comparable level in around 20–30% of cases of those disease entities.

#### 4.6.1. THP-1

The THP-1 was established from the peripheral blood of a 1-year-old boy with AML [123]. This line is characterized by t(9;11) (p22;q23) and KMT2A/MLLT3(AF9) fusion transcript and p-53 deficiency [37,61]. The THP-1 immortalized cells resemble primary monocytes and macrophages regarding morphology and differentiation properties. The THP-1 cells in the monocyte state can differentiate into macrophages after exposure to TPA, VD3, TNFα, IFN-γ, and retinoic acid [124]. The presence of the *PICALM*/*MLLT10* (AF10) fusion gene and t(10;11) (p12;q14) was confirmed in the THP-1 [37].

The THP-1 was commonly used in a variety of experiments. Since isolation, it has been the most popular cell line in research regarding immune and inflammatory response mechanisms. The advantage of THP-1, unlike human primary monocytes or macrophages, is a homogeneous genetic background, which reduces the degree of cell phenotype variability [125].

Cells are also susceptible to genetic modifications using siRNAs, which allows for reducing the expression of selected genes efficiently. The THP-1 line has been used for numerous studies to understand the immune response mechanism to the antigen and new potential therapies for inflammation [126]. The evaluation of the modulation of inflammation by food compounds, e.g., quercetin, citrus pectin, and barley glucan, was also carried out [127]. The THP-1 and the U-937 human cell line activation test (h-CLAT) method was developed, which can be used as a test model for skin sensitization [128,129]. Moreover, the THP-1 cells have been proven to produce enzyme-active lysozyme in increasing amounts in time [130]. Therefore, the line is used to study the effect of selected compounds, e.g., saponins or microbial preparations on the production and release of lysozyme activity in the monocyte/macrophage model [131,132].

Studies using the THP-1 activated by LPS (lipopolysaccharide) have shown the anti-inflammatory effect of essential oil derived from *Thymus vulgaris* L. by inhibiting the synthesis of pro-inflammatory cytokines IL-6, IL-8, IL-1β, and TNF-α [133]. However, the THP-1 is not a perfect model to study the response to LPS and its effect on IL-8 activity [134]. This line shows less sensitivity to LPS than primary monocytes. This is associated with reduced expression of CD14 in the cell line [135]. Stable transfection of the THP-1 cells with human CD14 increases the sensitivity of the cells to LPS. However, in primary monocytes, CAP37 (cationic antimicrobial protein) increases the LPS-induced IL-8 response approximately four-fold, which is not observed in the CD14-transfected THP-1 [134,136,137]. When choosing the THP-1 as a research model, it should also be considered that this line shows a reduced expression of IL-1β, IL-6, TNF-α, and CD68 than peripheral blood mononuclear cells (PBMCs) [135]. The THP-1 is reported to be useful in studying monocytic leukemia and the role of monocytes in immune response [123].

#### 4.6.2. U-937

The U-937 was established based on a pleural effusion of a 37-year-old man with generalized diffuse histiocytic lymphoma [138]. The U-937 carries *PICALM-MLLT10* fusion and mutated phosphatase and tensin homolog (*PTEN*), protein tyrosine phosphatase non-receptor type 11 (*PTPN11*), and *WT1* [80,139,140]. Similar to the THP-1, the U-937 is widely used as an experimental model to elucidate the mechanisms of monocytes and macrophage differentiation. However, these lines differ significantly in cell origin and maturity. The U-937 line is of tissue origin, so the cells are at a more mature stage, while the THP-1 cells are at a less mature stage [141].

The U-937 line was used to determine long-term macrophage changes associated with microgravity [142]. The U-937 immature cells can be stimulated to differentiate into macrophages by exposure to ATRA, VD3, PMA, TNFα, and IFN-γ [143,144,145,146,147]. PMA increases the expression of CD11b and CD36 differentiation markers and ROS production in the U-937 cells. These cells have been well characterized in their response to oxidative stress. Therefore, they are often used as a model of response to ROS [148,149,150,151,152,153]. Additionally, research showed that the differentiation of the U-937 cells into monocytes is most likely accompanied by the formation of ROS [154]. The U-937 cells express the Fas antigen and are sensitive to TNF and anti-Fas antibodies. Therefore, they are widely used in cell cycle and apoptosis studies [155,156,157]. They are also used to evaluate new anticancer drugs and as an in vitro test system to identify contact sensitizers. The U-937 was also used to prove the use of gallic acid (3,4,5-trihydroxybenzoic acid) as a potential chemotherapeutic agent against lymphoma. The exposure of the U-937 to gallic acid inhibited cell viability and induced apoptosis [158]. Other studies using this line have shown that agaritine, a component of *Agaricus* blazei *Murrill*, induced apoptosis of the U937 by activating caspase, releasing cytochrome c from the mitochondria [159]. The U-937 is widely used in cytotoxicity studies [160,161]. Also, to study skin sensitization, a test model called U-SENS™ was developed using the U-937 [162]. The U-937 is a suitable in vitro model for the HLA class I-restricted presentation of mycobacterial antigen in T cells [163].

A subline of the U-937 described as the U-937V shows increased sensitivity to TNFα cytocidal activity compared to U-937. Dying U-937 cells produce apoptotic bodies, while the U-937V shows no cell lysis. The U-937V subline was proposed as a good model for research on the mechanisms regulating the processes of cellular disintegration during apoptosis [164,165]. However, this variant of the U-937 line is not very widely used in research. The U-937 has been reported to be contaminated with the K-562 in stock from the suppliers. Still, the problem was corrected, and now stocks are confirmed to carry only the U-937 specific genotype and phenotype [166].

#### 4.6.3. MOLM-13 and MV4-11

The MOLM-13 was established from the peripheral blood of a 20-year-old man with M5a at the time of relapse after an initial myelodysplastic syndrome [167]. The MV4-11 was established from the blast cells of a 10-year-old male with biphenotypic B myelomonocytic leukemia [168]. The MV4-11 carries t(4;11) (q21;q23), typically observed in biphenotypic congenital leukemias and +8, +9 [168]. The presence of t(4;11) typically characterizes a subgroup of acute childhood leukemias, suggesting potential application in studying these disease entities [168,169,170]. Moreover, the *KMT2A*/MLLT2 (AF4) mutation was confirmed in the MV4-11 [37].

The MOLM-13 and the MV4-11 are *FLT3*-ITD positive cells that may reflect anticancer activity compounds against *FLT3*-mutated cells. Additionally, both lines are wild-type *TP53* [171]. Therefore, the MOLM-13 and the MV4-11 have been used as research models to understand the mechanisms underlying *FLT3*-ITD positive AML maintenance and drug resistance to tyrosine kinase inhibitors (TKI) treatment. Researchers demonstrated that *FLT3* inhibition induces the upregulation of *HDAC8* via forkhead box protein O1 (FOXO1) and forkhead box protein O3 (FOXO3)-mediated transactivation in AML cells with *FLT3*-ITD. Upregulated *HDAC8* by deacetylation inactivates p53 leading to leukemia maintenance and drug resistance during TKI treatment. HDAC9 inhibition significantly sensitizes *FLT3*-ITD-positive AML cells to TKI treatment. The use of the MV4-11 xenograft model also confirmed this [172]. In other studies using both lines, it was shown that the use of both *FLT3* inhibitors and human double minute 2 (HDM2) antagonists can be effectively applied in *FLT3*-ITD-positive AML therapy [173,174]. The MOLM-13 and the MV4-11 lines have been used in many studies leading to the discovery of selective FLT3 inhibitors [175,176,177], e.g., the LT-171-861 recently discovered and selected for clinical trials in AML patients with *FLT3*-ITD mutations [178]. The MOLM-13 and the MV4-11 were also used to create the cell line-derived xenograft (CDX)—the xenograft mouse model used in various research, mainly to test the efficacy of inhibitors *FLT3*. Pacritinib (SB1518), a TKI with potent *FLT3* and *JAK2* inhibitory activity, effectively reduced tumor growth in the MOLM-13 mouse xenograft model [179]. Other studies have shown the involvement of gilteritinib (an FLT3/AXL inhibitor) in blocking mutated *FLT3* in cell and animal models of AML. Treatment with gilteritinib in MV4-11 CDX mice significantly reduced *FLT3* activity and resulted in tumor regression [180].

### 4.7. Acute Erythroid Leukemia (M6)

Acute erythroid leukemia is a rare and uncommon subtype of AML, affecting primarily older patients and comprising less than 5% of all cases of AML. The most common mutations in AML-M6 include *NPM1*, transcription factors (e.g., *RUNX1*), *TP53*, splicing factors, and/or chromatin modifiers (e.g., *ASXL1*, *SRSF2*, *U2AF1*) [181]. In addition, chromosomal abnormalities are frequently observed in chromosomes 5 and 7 and less frequently in chromosomes 8, 16, and 21 [182].

Many M6 cell lines require external stimulation to proliferate. Several cell lines, including the AS-E2, HEL, K-562, OCIM1, and UT-7, express EPO receptors and are EPO-dependent. The AS-E2, F-36P, TF-1, and UT-7, are cultured as factor-dependent and undergo apoptosis without cytokines. The F-36P, TF-1, and UT-7 are constitutively cultured as factor-dependent cells, while others, such as the AS-E2, also depend on other cytokines such as GM-CSF and IL-3 [47]. It is important to note that other cytokines, such as IL-1B, also play a critical role in the growth and survival of these cells.

#### 4.7.1. HEL

The HEL cell line is an erythroblast cell line derived from the bone marrow of a 30-year-old Caucasian male patient. It has been shown to differentiate randomly into erythroblast-like cells and has been used to study the differential expression of globins [183]. The HEL has also been found to be a *JAK2 V617F*-mutated cell line [184]. This mutation is commonly found in Ph1-negative patients and leads to the constant activation of the JAK/STAT pathway, resulting in increased cell proliferation and inhibited apoptosis. In addition, the HEL has been observed to exist in the form of two subclones: CKIT-positive, CD41b-negative (erythroid lineage), and CKIT-negative, CD41b-positive (megakaryocytic lineage) [185].

The HEL has been used as a model to study the modulation of epigenetic modification by curcumin. It has been shown that this agent dynamically modulates HDAC and HAT activity. The exposure of the HEL (and the K-562) cells to curcumin restore the cytokine signaling of negative suppression regulators of cytokine signaling 1 (SOCS1) and 3 (SOCS3) by inhibiting the overexpression of HDAC activity [184]. In addition, the inhibition of JAK1/2 kinase and downstream messengers of STAT3, STAT5, and ERK1/2 have been shown to decrease the viability of curcumin-resistant cells [186,187].

#### 4.7.2. OCI-M1

The OCI-M1 cell line was derived from leukemic blasts from a 62-year-old patient with chronic lymphocytic leukemia. These cells are characterized by their large size, large nuclei, and ability to form large multinucleated cells. In addition, OCI-M1 are peroxidase- and chloroacetate esterase-negative, PAS-positive, and NSE-positive. Additionally, the OCI-M1 cell line has more EPO receptors than other cell lines, with around 3000 receptors per cell. In contrast, cell lines such as HEL and K-562 have fewer than 250 EPO receptors per cell [188]. That makes the OCI-M1 a valuable model for studying EPO receptors. It has also been observed that inducing erythroid differentiation with DMSO increases the expression of EPO receptors. Interestingly, treatment with PMA decreases the number of receptors per cell from 2210 to 370 without altering receptor affinity.

#### 4.7.3. OCI-M2

The OCI-M2 is an erythroleukemic cell line derived from a 56-year-old patient with acute myeloid leukemia. The OCI-M2 is characterized by its small size, basophilic cytoplasm, and large nuclei, distinguishing it from the larger OCI-M1 cells. The OCI-M2 cell line can differentiate along the erythroid lineage or the myeloid/monocytic/megakaryocytic lineage when induced by PMA. Karyotype analysis has revealed that OCI-M2 cells carry several chromosomal abnormalities, including +6, +8, add(9) (p23), del(9) (p12p21), t(10;12) (p12;p12), del(17) (q11q21.1), +20, −21, and +3 mar. Moreover, the OCI-M2 cells ectopically express the *NKL* homeobox gene *NKX2-4*, which disrupts megakaryocyte and erythrocyte differentiation. The activation of NKX2-4 leads to the aberrant deregulation of several genes encoding megakaryocytic and erythroid differentiation processes, including *ETV2*, *HEY1*, *IRF6*, and *SOX7*, thereby promoting M6 development [189].

The OCI-M2 cell line has been used as a model in studies investigating resveratrol’s ability to inhibit the activation of transcription of nuclear factor NF-kB through the suppression of IL-1B [190]. It has also been used in viability, apoptosis, and PARP-cleavage induced by caspase 3 assays [191].

#### 4.7.4. F-36P

The F-36P is a cell line derived from the bone marrow of a 65-year-old patient with refractory anemia, excess blasts, and MDS [192]. These cells are positive for the leukocyte antigen CD45 and express CD13, CD33, CD34, and CD71. The F-36P and its sublines require external stimulation for long-term survival and growth. The parental line, F-36P, requires GM-CSF or IL-3 for continuous growth, while the subline, F-36E, can be sustained in EPO alone. When GM-CSF and IL-3 are reduced, cells die within a few days, even in a medium supplemented with FCS.

The F-36E subline, in the presence of EPO, can be induced to synthesize hemoglobin at a significant level constitutively. Interestingly, the parental F-36P line can also be induced to synthesize hemoglobin if GM-CSF or IL-3 are replaced with EPO [192]. Therefore, this cell line is useful for studying the survival and growth of cells dependent on GM-CSF, IL-3, and other cytokines [193].

#### 4.7.5. TF-1

The TF-1 is a cell line derived from the bone marrow of a 35-year-old patient with erythroleukemia who suffered from severe pancytopenia [194,195]. The TF-1 cells carry a homogenous chromosomal abnormality (54, X) and are growth-dependent on EPO, GM-CSF, or IL-3. They also respond to several hematopoietic growth cytokines, including IFN-γ, IL-4, IL-5, IL-6, IL-13, LIF, NGF, OSM, SCF, and TNF-α [194]. The TF-1 cells constitutively express globin genes, indicating their commitment to the erythroid lineage, although they do not express glycophorin A or carbonyl anhydrase I. Hemoglobin synthesis in these cells can be induced with hemin and delta-aminolevulinic acid, while TPA leads to the rapid differentiation of the TF-1 cells into macrophage-like cells [195]. Although EPO sustains short-term growth in TF-1 cells, it does not induce differentiation. Specifically, this cell line is an immature erythroid line that can differentiate into more mature erythroid cells or macrophage-like cells in the presence of GM-CSF, IL-3, or EPO. TF-1 cells have also been used to study the effect of GM-CSF and IL-3 on proliferation in the context of TNFR2 expression, the induction of STAT5 phosphorylation by IL-3, and the biological activities of the anti-IL-6R nanobody ALX-0061 [196,197,198].

A subclone of the TF-1 called a TF1a has also been described. The TF-1a is a variant of the TF-1 cell line that is not dependent on growth factors for its growth. These cells can respond to various cytokines but with a different response pattern than the parental cell line. In contrast to the TF-1 cells, the TF-1a cells can form colonies in soft agar when cultured with the addition of growth factors and produce invasive tumors in nude mice. In addition, there is a slight, continuous activation of MAP kinase and MEK proteins in TF-1a cells but not in TF-1 cells. Regarding their phenotype, TF-1 cells are positive for CD34 and CD38 markers, while TF-1a cells are only positive for CD34. Additionally, TF-1a cells are resistant to TNF-alpha-induced apoptosis, unlike TF-1 cells. Overall, TF-1a is a valuable model for studying human primitive myeloid progenitor cells and examining the process of myeloid cell transformation into a more malignant state. It can also study signaling pathways involved in spontaneous and growth factor-induced cell growth.

#### 4.7.6. AS-E2

The AS-E2 cell line is derived from the bone marrow of a 62-year-old patient with pure erythroid leukemia. AS-E2 show near-tetraploid karyotype 67–82, XXYY, del(1) (p32p36), −2X2, del(3) (p21), del(3) (q21), −4, der(4)t(1;4) (q21;p16)X2, −5, add(8) (p23), add(8) (q24)X2, add(9) (q34), +del(9) (q11q22), −10X2, add(10) (p14), add(11) (q23), i(11) (q10), −13X2, +14, −15X2, add(15) (p12), −16X2, −17, −18, −19X2, −22X2 [199]. The AS-E2 cells display properties of late erythroid progenitor cells and are the only cells in the M66 panel that require EPO for their survival and growth [47]. The AS-E2 are reported to be useful in studying the effect of EPO on the proliferation of leukemic cells because they do not respond to any cytokines supporting the growth of erythroid lineage cells, except EPO [199]. In the presence of other cytokines, such as interleukin-3 or GM-CSF, the growth of the AS-E2 cells is inhibited. These cells also have low affinity for EPO receptors on their surface and are positive for CD36, glycophorin A, and CD71 but negative for CD41. Interestingly, the AS-E2 cells express globin transcription factor 1 (GATA-1), also known as the erythroid transcription factor. GATA1 plays a crucial role in regulating the expression of genes involved in the development of red blood cells, particularly in the maturation of precursor cells and platelets, as well as in proper cell formation [200]. The AS-E2 cell line has been used as an EPO-dependent model in studies related to the survival and proliferation of cells. In addition, the cell line seems to be a useful tool in determining the influence of GATA-1 gene expression [199].

### 4.8. Acute Megakaryoblastic Leukemia (M7)

The M7, like the M6, is an unusual subtype of AML, representing 3–5% of all cases. It affects children with Down’s syndrome (approximately 50%) rather than adults, occurring in 1% [201,202]. In normal conditions, megakaryoblasts are the most immature precursor cells, which form platelet lineage cells essential for proper blood clotting, growing to promegakaryocytes and further to megakaryocytes. However, in the M7, the malignant megakaryoblasts account for the predominant proliferative cells leading to the destruction of organs and tissues. Besides, extensive myelofibrosis is frequently accompanied by bone marrow biopsy [203]. M7, in the WHO 2016 classification, has been divided into three categories: the M7-Down syndrome pediatrics group, expressing *GATA1* mutations, followed by chromatin regulator mutations, for instance, *EZH2* or signaling molecules such as those in the *JAK*/*STAT* and *RAS* pathways [204,205]. Subsequently, non-Down-syndrome M7 pediatric group has been described with mutation in chromosome t(1;22) (p13;q13) *RBM15-MKL1*, and *CBFA2T3-GLIS2*, *NUP98-KDM5A* mutations [206]. Next, adults with M7 commonly have lost chromosomal translocations, and they exhibit *TP53*, *DNMT3A*, and *RB1* mutations in genes in the cohesin, splicing factor, and *ASXL* families [207]. Moreover, M7 expresses CD34, CD4 markers, characteristic myeloid markers, and megakaryocytic markers (Table 1). Since M7 is an aggressive AML form, all patients’ prognosis becomes very poor.

#### 4.8.1. CMK

The CMK megakaryoblastic and hypotetraploid cell line was established from the peripheral blood of an infant with Down’s syndrome and acute megakaryocytic leukemia with translocation der(17)t(11:17) [37,208,209]. In the CMK, no expression of p53 mRNA was found [80]. The leukemic cell line demonstrates *JAK2 V617F* tyrosine kinase mutation. The CMK cells are distinguished by one or more round/ovoid nuclei with one or more prominent nucleoli, and they are composed of basophilic cytoplasm with protrusions [209]. The CMK cells are characterized by megakaryocytic features of lineage since they display glycoprotein GP Ilb/Illa and platelet peroxidase activity, C and glycophorin A. Particularly, this cell line with megakaryocytic features also expresses erythroid and myeloid markers (Table 1). Moreover, the studies demonstrated that IL-3 and GM-CSF induced the proliferation of CMK cell line-CSF. In addition, it was shown that phorbol esters inhibited CMK proliferation and stimulated morphological change, which resulted in cytoplasm dissociation into numerous segments. Furthermore, phorbol esters caused the induction of GPllb/Iila antigen expression, whereas they lessened the expression of glycophorin A. Therefore, using CMK cells may be considered in the studies concerning the estimation of megakaryocyte platelet formation of specific proteins. The described cell line should be studied for distinguishing the basis of the afferent association between megakaryoblastic leukemia and Down’s syndrome, as well as for the further study of megakaryocytic differentiation. They appear to be the most suitable model for studies in megakaryocytopoiesis [209].

#### 4.8.2. ELF-153

The ELF-153 cell line was established from a 41-year-old patient with AML during refractory relapse, demonstrating the progression of acute myelofibrosis [210]. The ELF-153 cells may occur in two morphological populations. The major one consists of small size marrow blast cells, and the other with large and mature megakaryocytes with multilobulated nuclei. Thus, the ELF-153 cells are the largest blast cells. In particular, major cells express blast morphology with myeloid markers phenotype (Table 1). The proliferation of ELF-153 was dependent on IL-3, GM-CSF, and IL-6. However, the cells were not stimulated by EPO, IL-7, IL-11, GC-SF, or FGF [210].

It was demonstrated that the ELF-153 was distinguished into three separate cell populations using flow cytometry (CD34+/CD61−, CD34+/CD61+, and CD34−/CD61+), resulting in various proliferative and endomitotic properties associated with different stages of megakaryoblastic differentiation. Moreover, the CD61+ cells displayed elevated GATA 1 and GATA 2 transcription factors. Thus, the ELF-153 may be considered a model for studying the molecular regulation of specific MK genes [189,210].

#### 4.8.3. UT-7

Derived from a 64-year-old man with acute megakaryoblastic leukemia, the UT-7 cell line is pluripotent, strictly cytokine-dependent, and dependent on growth factors [208]. These factors include IL-3, GM-CSF, and EPO. Furthermore, the described cell line possesses an enormous number of binding sites of EPO (7200 to 13,000/cell), which enables the investigation of EPO expression and receptor function. Due to this fact, a phenotype of the UT-7 subline, the UT-7/Epo, was examined. The UT-7/Epo was completely EPO-dependent. The studies showed that the UT-7/Epo stimulated tyrosine phosphorylation and induced the activation of p21, where the protein interferes with mitogenic signals of tyrosine kinase correlation. In addition, the UT-7/Epo affected the transcription factor GATA 1, one of the most significant proteins associated with activating erythroid, mast, and megakaryocyte lineage. The UT-7/Epo, similarly, differentiated to megakaryocytes in the presence of phorbol esters. Therefore, the UT-7 and its subline, the UT-7/Epo, may be the most suitable model for investigating EPO-growth-dependent cell lines and GATA 1 and p21-activation signaling pathway [208,211].

#### 4.8.4. M-07

The M-07 is a human leukemic cell line established from the peripheral blood of a baby with M7. The cell line expresses surface membrane platelet glycoproteins as well as early markers of hematopoietic differentiation. It is defined by a high sensitivity to the IL-3 growth-promoting activity [212,213].

The M-07e is a subline of the M-07, which is interleukin-3 and GM-CSF dependent [214]. The M-07e does not grow in the presence of other cytokines. However, it responds to IL-2, IL-4, IL-6, IL-9, and IFN-α, IF-β, and *IFN*-*γ*. This line was used as a model to study the IL-3 growth-dependent effect [212]. Both cell lines may be analyzed in the context of the factor-responsive leukemia cell lines and for identifying cytokine effects [193].

#### 4.8.5. MEG-01

The MEG-01 cell line was derived from the bone marrow of a 55-year-old patient with Ph1, who suffered from CML in a megakaryocytic blast crisis. The cell line carries the fusion gene *BCR*/*ABL* resulting from the Ph1 chromosome balanced translocation t(9;22) [215,216,217]. Moreover, the MEG-01 cell line is the first cell line described in the literature that can form platelet-like particles [218]. The MEG-01 cell line may differentiate into megakaryocytes in response to phorbol esters or valproic acid [219]. Therefore, the MEG-01 cell line has been used as a suitable model for studying human megakaryocytic maturation and differentiation [219].

#### 4.8.6. MEGAL

Cell line derived from AML7 [220]. Little is known about the MEGAL cell line. However, it expresses the *SET-NUP214* fusion gene due to a cryptic deletion del(9) (q34.11q34.13) [221]. Moreover, the MEGAL cell line is one of the unique cell lines identified with this mutation. Hence the described cell line seems to be a promising model for studying the *SET-NUP214* system and exploring its function [221].

### 4.9. Cell-Line Markers

Planning experiments with AML cell lines involves knowledge of ectopic markers associated with cells. As markers are essential for any researcher, cell line-specific markers are shown in Table 1.

**Table 1:** Use of AML cell lines with a list of expression markers based on data acquired from DSMZ (German Collection of Microorganisms and Cell Cultures), DSMZ CellDive database, and Cellosaurus database [191,222,223].

Disease	Cell Line	Markers	Use
M0	Kasumi-3	CD2−, cy/smCD3−, CD4+, CD5−, CD7+, CD8−, CD13+, CD14+, CD15−, CD19−, CD20−, CD22+, CD25+, CD33+, CD34+,CD38+, CD56+, cyCD68+, HLA-DR+, c-Kit+	*EVI1* and BET inhibitors research;
			Drug response in AML;
			Engraftment studies
	MOLM-16	CD3−, CD9+, CD13+, CD19−, CD22+ CD31+, CD33+, CD34+, CD36+, CD38+, CD41+, CD47+, CD56+, CD61+, CD62P+, CD63+, CD71+, CD110+, CD117+, CD119+ CD151+, CD235A+, thrombospondin+, vWf+, fibrinogen+, HLA-DR-	t(6;8) (q21;q24.3) model;
			PIM/FLT3 signaling;
			JAK2 V617F function research;
			*PMS2* and *RSPH10B2* deletion
M1	CTS	CD1+, CD2+, CD3+, CD4+, CD5+, CD7+, CD8+, CD10+, CD13+, CD14+, CD19+, CD20+, CD25+, CD33+, CD34+, HLA-DR+, D2-10+, P2+, HPCA-1+	t(6;11) (q27;q23) model;
			*KMT2*/*AF6* research model;
			GM-CSF and G-CSF differentiation;
			Pluripotent stem cell research
	UoC-M1	CD7+, CD24+, CD34+, CD38+, CD45+, HLA-DR+ CD61+	Monosomy 7 and 5q loss model;
			High *KMT2A* mRNA level
	KG1	CD3−, CD13+, CD14−, CD15+, CD19−, CD33+, CD34+, HLA-DR+	Cell maturation studies;
			*KMT2A* and WT *DNMT3A* research;
			Toxicology and drug testing;
			Macrophage differentiation;
	K-562	CD3−, CD14−, CD15+, CD19−, CD33+, CD71+, CD235a+	BCR-ABL1 fusion, Ph1 chromosome;
			Platelet-formation;
			p53-deficient
M2	Kasumi-1	CD3−, CD4+, CD13+, CD14−, CD15+, CD19−, CD33+, CD34+, CD38+, CD71+, HLA-DR+	t(8;21) model;
			*RUNX1-RUNX1T1* fusion research;
			*c-kit*, *TP53* mutations;
			Granulocytic and macrophage differentiation;
			Il-5 and TPA-induced differentiation;
	Kasumi-6	CD3−, CD4−, CD13+, CD14−, CD19−, CD33+, CD34−, cyCD68−, HLA-DR+	*FLT3*, *CEBPA*, *TP53* mutations;
			Model for differentiation research
			TPA-induced differentiation
	SKNO-1	CD3−, CD4+, CD13+, CD14−, CD15−, CD19-, CD33+ CD1, CD2, CD3, CD4, CD5, CD6, CD7, CD8, CD11, CD13, CD14, CD15, CD19, CD20, CD33, CD34, HLA-DR	t(8;21) (q22;q22) research;
			*RUNX1*/*RUNX1T1* fusion;
			Myeloid leukemogenesis studies;
	HL-60	CD3−, CD4+, CD13+, CD14−, CD15+, CD19−, CD33+, CD34−, HLA-DR-	t(15;17) model;
			Granulocytic and mononuclear maturation; Chemotherapeutics influence;
			Proliferation, apoptosis, and cell cycle study;
			Chemotactic response;
			miRNA studies
	PLB-985	CD3−, CD4+, CD13+, CD14−, CD15+, CD19−, CD33+, CD34−	Granulocytic, monocytic, macrophage maturation, proliferation;
			Neutrophil differentiation;
			Maturation studies of cells in early stage
M3	NB4	CD3−, CD4+, CD11b−, CD13+, CD14−, CD15+, CD19−, CD33+, CD34−, CD38+, HLA-DR-	ATRA resistance mechanisms
			*PML-RARA*Pro900Ser mutation;
			Retinoic acid, DMSO, TPA differentiation;
			Drug screening
	PL-21	CD3−, CD4 (+), CD14−, CD15+, CD19−, CD33+, cyCD68+, HLA-DR-	Lack of t(15;17)
			*KRAS*, *FLT3* mutations and WT *P53*;
			Kinase inhibitors studies
	UF-1	CD3−, CD4−, CD5−, CD8−, CD11b−, CD10−, CD7+, CD13+, CD19−, CD20−, CD33+, CD34−, CD38+, CD41− [82]	WT *RARA*;
			*PML-RARA* research;
			ATRA-resistance studies;
			Multi-drug screening with ATRA
	HT93	CD3−, CD19−, CD33+, CD34+, cyCD68+, HLA-DR−	t(15;17) and t(1;12) model with *PML-RARA* and
			*ETV6-ABL2 fusion*;
			*TP53* mutation;
			Differentiation, proliferation, cytokine studies
	AP-1060	CD3−, CD14−, CD15+, CD19−, CD33+, cyCD68+, HLA-DR−	t(15;17) and unique t(3;14) model;
			*PML-RARA*Pro900Leu mutation;
			ATRA and ATO resistance;
			Cytokine-dependent growth research;
			*ETV6-NTRK3* fusion model;
			Neutrophil maturation
M4	OCI-AML2	CD3−, CD4+, CD13+, CD15+, CD19−, CD33+, CD34−, cyCD68+, HLA-DR+	Mutated DNMT3A role in leukemogenesis;
			xenograft models
	OCI-AML3	CD3−, CD4+, CD13+, CD14−, CD15+, CD19−, cyCD68+, HLA-DR-	Mutated DNMT3A role in leukemogenesis; xenograft models;
			*NPM1* mutation;
	MUTZ-11	CD4+, CD7+, CD13+, CD15+, CD33+, CD65+, CD68+ [102]	Response to cytokines;
			Dendritic cell myeloid differentiation;
			*KMT2A* and *FLT3* mutations
	MUTZ-8	CD3−, CD4−, CD13+, CD19−, CD33+, CD34+, HLA-DR+	t(5;11) model;
			*JAK2 V617F* mutation;
			Cytokine response
	MUTZ-3	CD3−, CD4+, CD5−, CD7−, CD8−, CD13+, CD14+, CD15+, CD19−, CD34+, HLA-DR+	Role of FLT3 in leukemia pathogenesis; Cytokine response;
			Dendritic cell myeloid differentiation
M5	THP-1	CD3−, CD4+, CD13+, CD15+, CD19−, CD34−, cyCD68+, HLA-DR+	t(9;11) (p22;q23) with *KMT2A*/*MLLT3*(AF9) fusion model;
			Immune and inflammatory response; inflammation;
			Susceptible to genetic modifications;
			Skin sensitization model;
			Cytokine response;
	U-937	CD3−, CD4+, CD14−, CD15+, CD19−, CD33+, CD34−, CD54+	Monocyte and macrophage differentiation; Response to ROS;
			Skin sensitization model;
			Model for cell apoptotic disintegration
	MOLM-13	CD3−, CD4+, CD14−, CD15+, CD19−, CD33+, CD34−, CD68+, HLA-DR−	Drug resistance research; Leukemia xenograft models;
			WT *TP53*, *FLT3*-ITD+ in AML
	MV4-11	CD3−, CD4+, CD5−, CD8−, CD10−, CD14−, CD15+, CD19−, CD33+, CD34−	t(4;11) (q21;q23) model;
			Mechanisms of FLT3-ITD+ AML Leukemia; Xenograft models;
			WT *TP53, FLT3*-ITD+ in AML
M6	HEL	CD3−, CD13+, CD14−, CD19−, CD33+, CD41a+, CD71+, CD235a+	*JAK/STAT* signaling pathway,
			Differential globins expression
			*JAK2* mutation
	OCI-M1	CD3−, CD4−, CD15+, CD19−, CD33+, CD34−, CD41−, CD42−, CD71+, HLA-DR+	Model for the EPO receptors studies,
	OCI-M2	CD3+, CD14+, CD19−, CD33 (+), CD71+	Studying NFkB inhibitors;
			*NKX2-4* expression
	F-36P	CD3−, CD13+, CD14−, CD15−, CD19−, CD33+, CD34+, CD41−, CD42−, CD71+, CD235a+	Study of IL-3 and GM-CSF dependence;
			Primitive progenitor cell model;
			Myeloid cell differentiation;
			Oncogenesis
	TF-1	CD3−, CD13+, CD14−, CD15−, CD19−, CD33+, CD34+, CD71+, HLA-DR+	*TNFR2* expression;
			*TPA* macrophage differentiation;
			Cytokines response;
			Anti-IL-6R nanobody ALX-0061
	AS-E2	CD2−, CD3−, CD10−, CD11b+, CD13+, CD19−, CD25−, CD33+, CD36+, CD41−, Glycophorin A+, CD71+ [199]	Erythroid progenitor cells;
			EPO-dependent growth;
			*GATA-1* expression
M7	CMK	CD3−, CD13+, CD14−, CD15−, CD19−, CD33+, CD34+, CD71+, CD235a+	der(17)t(11:17) model;
			*JAK2 V617F* mutation;
			Megakaryocytopoiesis research;
	ELF-153	CD3−, CD4+, CD13+, CD14−, CD15−, CD19−, CD33+, CD34+, HLA-DR+	High GATA level;
			Megakaryocytopoiesis research
	UT-7	CD3−, CD13+, CD14−, CD15−, CD19−, CD33+, CD34−, cyCD68+	Response to cytokines;
			EPO-response;
			GATA expression,
	M-07	Non disclosed	Cytokine response
	M-07e	CD3−, CD13+, CD14−, CD19−, CD33+, HLA-DR-	Cytokine response
	MEG-01	CD3−, CD13+, CD15+, CD19−, CD33+	t(9;22) model;
			*BCR*/*CBL* fusion;
			Megakaryocytic differentiation/maturation
	MEGAL	CD3−, CD13−, CD14−, CD19−, CD33+, CD34+, CD71+, CD235a−	*SET-NUP214* fusion

### 4.10. Controls from Healthy Donors

Performing research regarding new AML drugs, inhibitors, etc., is often associated with a lack of proper healthy controls. Showing the absence of toxicity on non-malignant cell lines such as bone marrow cells is often crucial to proving the compound’s usefulness. As a healthy, physiological control, hTERT bone marrow transfected cells may be used, e.g., hTERT-immortalized mesenchymal stem cells or even patient-derived bone marrow mononuclear cells [224,225,226]. Yet, although hTERT-transformed cells are an excellent model for studying potential drugs’ cytotoxic influence, testing on healthy donor bone marrow cells is advised.

## 5. Conclusions

Although AML is a very heterogeneous disease, the established cell lines allow for successful, repetitive, and cost-effective examination of AML biology for decades. Various genetic aberrations carried by specific cell lines allow for successfully studying whole disease entities, AML subtypes, or single gene alterations. Many cell line models invented over the years are useful in leukemia cell differentiation, proliferation studies, as well as drug screening assays. The problem with established leukemia cell lines is often the lack of proper healthy control, which can be overcome using patient bone marrow samples or hTERT-transformed bone marrow cells.

## Data Availability

Not applicable.
